# Treatment of Chronic Myelomonocytic Leukemia with 5-Azacytidine: Case Reports

**DOI:** 10.1155/2012/369086

**Published:** 2012-05-28

**Authors:** Peter Rohon, Jana Vondrakova, Anna Jonasova, Milena Holzerova, Marie Jarosova, Karel Indrak

**Affiliations:** ^1^Department of Hemato-Oncology, Faculty of Medicine and Dentistry, Palacky' University, Olomouc, IP Pavlova 6, 775 20 Olomouc, Czech Republic; ^2^First Department of Medicine, Charles University General Hospital, U nemocnice 2, 128 08 Prague, Czech Republic

## Abstract

Epigenetic therapy with hypomethylating agent (5-azacytidine; AZA) is common in the management of specific subtypes of myelodysplastic syndrome (MDS), but there are only few studies in chronic myelomonocytic leukemia (CMML) patients. In this paper our experience with 3 CMML patients treated with AZA is described. In one patient transfusion independency was observed after 4 treatment cycles; in one case a partial response was recorded, but a progression to acute myeloid leukemia (AML) after 13 AZA cycles has appeared. In one patient, AZA in reduced dosage was administered as a bridging treatment before allogeneic stem cell transplantation (ASCT), but in the control bone marrow aspirate (before ASCT) a progression to AML was recorded. Future studies are mandatory for evaluation of new molecular and clinical features which could predict the efficiency of hypomethylating agents in CMML therapy with respect to overall survival, event-free survival, quality-adjusted life year, and pharmacoeconomy.

## 1. Introduction

Chronic myelomonocytic leukemia (CMML) is a clonal disorder of hematopoietic stem cell characterized by monocytosis (>1 × 10^9^/L) in the peripheral blood, absence of the Philadelphia chromosome or *BCR/ABL*1 fusion gene, fewer than 20% blasts and one or more lineages showing dysplastic features. It occurs often in elderly patients (>70 years) and predominantly in men [1]. In 80% of cases CMML arises *de novo*, in 20% from prior myelodysplasia occasionally with monocytosis. Splenomegaly is observed in 30–50% of patients with rare rupture, hepatomegaly in 20% of cases [2]. 

 In the new WHO 2008 classification of tumors of hematopoietic and lymphoid tissues, CMML was reclassified as a myelodysplastic/myeloproliferative disorder characterized by a proliferation of the myeloid lineage and by a dysplastic erythropoiesis; it was divided in two subclasses according to peripheral blood and bone marrow blast count: CMML-1: <5% blasts and <10% blasts in peripheral blood and bone marrow, respectively, and CMML-2: 5–19% blasts (or Auers' rods) and 10–19% blasts in peripheral blood and bone marrow, respectively [3]. Cytochemical staining for naphthyl-butyrate esterase highlights monocytic elements. Cytogenetic abnormalities can be confirmed in 20–40% of CMML cases including trisomy 8, monosomy 7, and 7q-, abnormalities of 12p; *RAS* mutations are observed in 30% and *JAK2 V617F* mutations in 13% of the patients [4, 5].

 CMML treatment is very arduous and significantly influenced by patients' age, prognosis is variable with a median survival of about 19 months, range 12–24 months (NCI 2010). Patients are usually treated with transfusions (supportive care), in the minority of them cytoreduction with hydroxyurea or cytarabine can be used, allogeneic stem cell transplantation (ASCT) is reserved for a limited number of younger patients only [6]. Epigenetic therapy with hypomethylating agents (5-azacytidine; AZA and decitabine) has activity in the myelodysplastic syndrome (MDS) and has also received approval for the treatment of CMML. The specific efficacy in CMML has not been studied yet in a larger cohort of patients [6–8]. AZA is incorporated into RNA and reaches DNA following reduction by ribonucleotide reductase. AZA and also 2-deoxy-5-AZA (decitabine) decrease activity of DNA methyltransferase (DNMT), reverting aberrant DNA methylation, and increasing the expression of silenced genes, leading to cellular differentiation and/or apoptosis [9, 10].

## 2. Case Reports

 3 CMML patients (2 men and 1 woman) were treated in our institution since 2010. Two patients were treated with AZA at 75 mg/m^2^ s.c. for 7 consecutive days monthly and one patient was treated with reduced regimen 100 mg s.c. for 5 consecutive days. Patients' characteristics are summarized in Table 1. AZA treatment was well tolerated with only mild cutaneous toxicity (localized erythema).

**Patient 1**

59-year-old man with severe comorbidities (history of pulmonary interstitial process, liver cirrhosis and esophageal varices, haemorrhagic gastropathy, and seropositive rheumatoid arthritis) was not considered to be a suitable candidate for ASCT. Erythropoiesis-stimulating protein (ESP) showed no effect (>10 weeks of administration). Transfusion dependency (TD) was 3 TU/months. After 4 cycles of AZA, a transfusion independency was achieved (lasting more than 8 weeks). Patient currently continues with the epigenetic therapy (6 cycles of AZA are planned). The overall survival is 21 months to the current date.

**Patient 2**

57-year-old woman with metabolic syndrome started the CMML treatment for monocytosis progression (6.3 × 10^9^/L, within 2 weeks) with hydroxyurea. Initial cytoreduction was complicated by septic shock (no etiologic agent was identified). Bridging therapy composed of AZA (reduced regimen, 100 mg s.c. for 5 consecutive days) and due to re-progression in monocyte count (11.2 × 10^9^/L), a cytarabine regimen (100 mg i.v. for 5 consecutive days) was administered before planned ASCT from HLA identical brother (procedure was postponed for significant internal comorbidities in brother). Recovery of megakaryopoiesis with stable platelet count (40–60 × 10^9^/L) (>8 weeks) was recorded, however patient has progressed to AML (60% myeloblasts: CD33+, CD13+, CD65+, HLA-DR+, CD117+, MPO+) before the ASCT. Patient is currently well with 100% donor chimerism at day +35 after ASCT.

**Patient 3**

**Figure 1:**
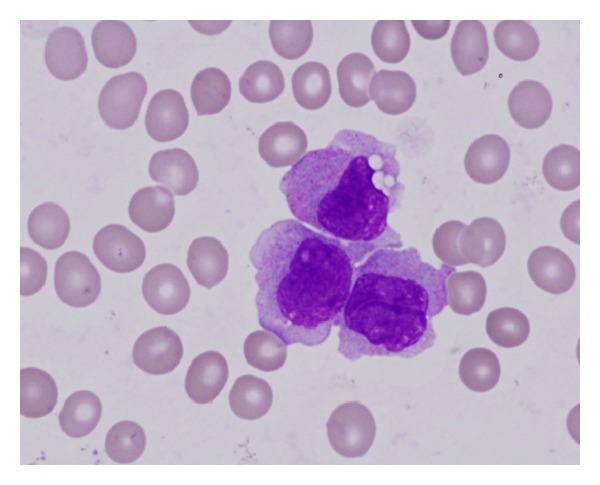
Bone marrow aspirate (×1000, panoptical staining) from the time of diagnosis; monocyte population (atypical monocytes, promonocytes). The finding was classified as CMML-2 (16% of myeloblasts).

**Figure 2:**
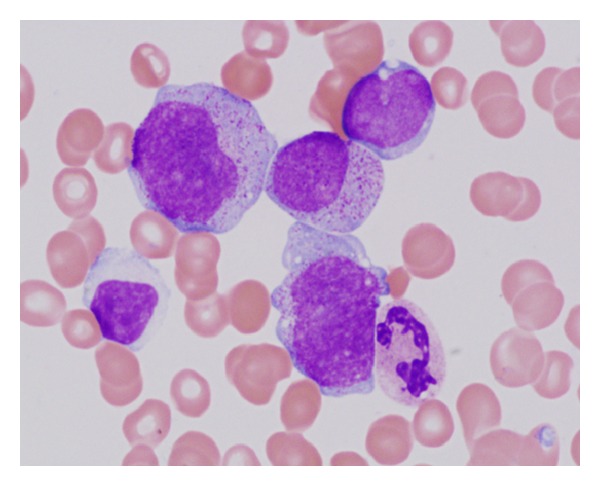
Bone marrow aspirate (×1000, panoptical staining) after 13 AZA cycles; myeloblasts and monoblasts, progression to AML (60% of myeloblasts).

72-year-old man with metabolic syndrome, ischemic heart disease, and bronchial asthma started the AZA therapy because of transfusion dependency (3 TU/months). After 4 cycles of AZA a partial response and a transfusion independency (for 6 months) was achieved. Stable peripheral blood count obtained during application of 13 AZA cycles. After 13 AZA cycles a progression to AML was described in the control bone marrow aspirate (Figures 1 and 2). The overall survival is 17 months to the current date.

## 3. Discussion and Conclusion

 Epigenetic regulation is influenced by modulation of gene expression without alteration of the coding sequence. Two complementary mechanisms support this regulation: methylation of DNA CpG islands by DNMT leading to silencing of the gene expression and the histone tails modifications which change the accessibility of the reading frame to RNA polymerases [11, 12]. Inhibition of DNMTs and incorporation of AZA into DNA are the key mechanisms of action and make its effect S-phase dependent [13]. AZA also modifies the function of T-regulatory cells and can inhibit hematopoiesis in patients with MDS [14]. Efficacy of AZA was confirmed in the treatment of MDS (especially in high risk patients). AZA in particular, significantly prolonged the median time of progression to acute myeloid leukemia or death and prolonged overall survival compared with conventional care regimen [15–17]. Hypomethylating agents are also used in CMML treatment and there are no prospective studies with sufficient numbers of patients. A retrospective analysis of 38 CMML treated with AZA at the dosage 75 mg/m^2^ for 7 consecutive days or 100 mg/m^2^ for 5 consecutive days monthly showed 39% overall response rate, with 11% CR, 3% PR and 25% HI (hematological improvement). The median response duration was 6.5 months [7]. The treatment of CMML with hypomethylating agents is still controversial. A lot of issues are under the discussion: the best treatment schedule [7], the number of treatment cycles, termination of the treatment after achieving of complete remission, and bridging therapy before ASCT. Moreover, the pharmacoeconomy is an important point of epigenetic therapy with respect to quality-adjusted life year. Future studies are mandatory for evaluation of new molecular and clinical features which could predict the efficiency of hypomethylating agents in CMML therapy.

## Figures and Tables

**Table 1:** Patients' characteristics.

	Patient 1	Patient 2	Patient 3
Basic information			

Age at dg. (years)	59	57	72
Sex	Male	Female	Male
CMML type	CMML-1	CMML-1	CMML-2
IPSS	LR	INT-1	INT-2
Cytogenetics	46, XY [21]	46, XX [20]	46, XY [18]
TD (TU/months)	3	—	3
ESP treatment	+	−	+
Dg.-AZA (months)	17	1	4
No. of AZA cycles	4	1	13

Counts at diagnosis			

Hb (g/L)	70	86	73
WBC (10^9^/L)	11.44	3.98	5.81
Monocytes (10^9^/L)	4.63	1.46	2.54
PLT (10^9^/L)	114	11	209
PB-blasts (%)	0	5	11

Counts (4 AZA cycles)			

Hb (g/L)	85	—	121
WBC (10^9^/L)	6.69	—	6.22
Monocytes (10^9^/L)	0.68	—	3.03
PLT (10^9^/L)	164	—	126
PB-blasts (%)	0	—	3

Comments			

	Transfusion independency (>8 weeks)	AZA-reduced, bridging treatment before ASCT → progression to AML on AZA therapy	13 cycles of AZA → progression to AML

AZA: 5-azacytidine (Vidaza, Celgene); IPSS: international prognostic scoring system; TD: transfusion dependency; TU: transfusion unit; ESP: erythropoiesis-stimulating protein; Hb: haemoglobin; WBC: white blood cells; PLT: platelets; PB-blasts: peripheral blood blast counts; ASCT: allogeneic stem cell transplantation.
